# Age-Dependent Developmental Response to Temperature: An Examination of the Rarely Tested Phenomenon in Two Species (Gypsy Moth (*Lymantria dispar*) and Winter Moth (*Operophtera brumata*))

**DOI:** 10.3390/insects9020041

**Published:** 2018-04-11

**Authors:** David R. Gray

**Affiliations:** Natural Resources Canada, Canadian Forest Service—Atlantic Forestry Centre, Fredericton, NB E3B 5P7, Canada; david.gray@canada.ca

**Keywords:** phenology, age dependence, age-dependent developmental rate, gypsy moth, winter moth, niche models, Jensen’s inequality

## Abstract

The pervading paradigm in insect phenology models is that the response to a given temperature does not vary within a life stage. The developmental rate functions that have been developed for general use, or for specific insects, have for the most part been temperature-dependent but not age-dependent, except where age is an ordinal variable designating the larval instar. Age dependence, where age is a continuous variable, is not often reported (or investigated), and is rarely included in phenology models. I provide a short review of the seldom-investigated phenomenon of age dependence in developmental response to temperature, and compare the derivation of the winter moth egg phenology model by Salis et al. to the derivation of another egg phenology model with age-dependent responses to temperature I discuss some probable reasons for the discrepancies (acknowledged by Salis et al.) between modelled and observed developmental rates of the winter moth, and discuss the contribution that geographically robust phenology models can make to estimates of species distributions.

## 1. Introduction

The history of phenological observations dates to at least the 11th C BCE [1]. Phenological studies remained principally limited to observations of phenological events until Réaumur [2] observed that differences between locations in phenological events could be explained by differences in the sum of daily temperatures between some arbitrary starting date to the date of the event. The developmental responses of organisms to temperature have been of interest since. Insect phenology models have been built as components of integrated pest management (IPM) programs [3] for more efficacious applications of pesticides and for optimizing biological control tactics [4]. Phenology models have been used in investigations of biological ranges of invading insects under current [5] and future [6] climate conditions, and in the investigation of biological invasions via international marine traffic [7,8]. Phenology models provide valuable insight into the potential disruption of the important synchrony between insect herbivores and their hosts under future climates [9].

In a recent paper, Salis et al. [10] examined the phenology of eggs of the winter moth (*Operophtera brumata* L.) and proposed a phenology model in which developmental response is dependent on the “interactive effects of temperature and developmental stage” (p. 1777). However, the authors examined only one stage—the egg stage—and what they actually observed was the far more interesting phenomenon of *age* dependence, where age (defined below) must be dealt with as a continuous variable within the egg stage: 0 (at oviposition) to 1 (at egg hatch). Experimental confirmation of the age-dependent phenomenon in a second species (after the gypsy moth, *Lymantria dispar dispar* [Linnaeus]) with a notoriously difficult-to-model egg stage [11,12,13,14] is important because it suggests that the phenomenon may be more common than we have acknowledged. Other phenology models may be improved by including an age effect. I will examine the age-dependence observed in the Salis et al. [10] experiment after establishing some basic terminology for the discussion.

### 1.1. Phenology Modeling: The Basics

*Phenology* is the study of periodic biological events as influenced by the environment—especially temperature, photoperiod, moisture, and nutrition [15,16]. Temperature is the dominant or sometimes sole environmental factor influencing phenology in many insects [17], and it is the only factor discussed here. Phenological studies have evolved over the centuries from the compilation of georeferenced dates of particular biological events to the development of mathematical models (phenology models) that predict the timing of a phenological event based on temperature inputs. In phenology studies, *developmental rate* (*r*) at time *t* is the velocity of progression toward the phenological event, and is a function of the temperature *T* at time *t*. The integral of developmental rate at time *t* is *age* (*A*). Thus:

(1) r(t)=f(T(t)),and

(2) $$A\left(t\right)=\int_{t=0}^{t}f\left(T\left[t\right]\right)\;dt.$$

The phenology model has a time step of *dt*, and the phenological event occurs at time *t* when A(t)=1. Thus, *age* is a measure of the accumulated to-date progress toward the phenological event, and is distinct from time in poikilotherms. *Stage* describes the morphological form (e.g., egg, larva) of insects, and is distinct from *age*.

### 1.2. Stage-Dependent Developmental Response to Temperature

In phenology modeling, the *developmental rate function* describes the relationship between *r* and *T*:

(3) r=f(T).

The earliest form of the developmental rate function (Equation (3)) was devised by Réaumur in a six volume series (1734–1742, reviewed by Egerton [18]). His linear heat summation model is now commonly known as a degree-day model:

(4) r(T)=1DD×T+m,

where *DD* is the thermal constant (i.e., the requisite number of degree-days (above a minimum temperature *T*_min_) for the phenological event to occur) and *m* is the *y*-axis intercept (=−TminDD). Nonlinearity in the r=f(T) relationship (Equation (3)) was observed at least as early as 1932 [19], and the advent of digital desktop computers was accompanied by the use of non-linear functions such as (but not limited to) the “reduced Sharpe-Schoolfield” [20,21] model used by Salis et al. [10] for the developmental rate function:

(5) $$r\left(T\right)=\frac{\frac{\rho T}{T_{ref}}\times \exp\left[\frac{H_{A}}{R}\left(T_{ref}^{-1}-T^{-1}\right)\right]}{1+\exp\left[\frac{H_{L}}{R}\left(T_{L}^{-1}-T^{-1}\right)\right]}.$$

Regardless of whether a linear or a nonlinear function is used, the underlying assumption of phenology modeling has been that the developmental rate function (Equation (3)) does not change for the duration (*D*) of the developmental stage(s) under consideration. Thus, a given temperature *T* will always cause the same developmental rate *r*. This forms the basis for traditional experiments to determine the developmental rate function (Equation (3)), because rate (*r*) at temperature *T* is the inverse of the duration (*D*) required to complete development at the constant temperature *T*:

(6) r(T)=1D(T).

Duration is measured in days (*d*), and rate is 1d. Sanderson [22] cites several examples of experiments from the very early 1900s wherein researchers noted differences among life stages in the required “temperature accumulation” for development. It is now generally assumed that a given developmental rate function is applicable to only one particular life stage.

### 1.3. Age-Dependent Developmental Response to Temperature within a Life Stage

More than a century ago, Sanderson and Peairs [23] observed an age-dependent developmental response in laboratory experiments with the egg stage of gypsy moth (then *Porthetria dispar* L.), although they did not yet use the term “age”. Logan et al. [24] tested for age independence in the developmental rate function of the larval stage of gypsy moth (*Lymantria dispar dispar* L.). They found statistical differences among the instars in their developmental responses to temperature. A straight-forward approach to accounting for age dependence in the larval stage is to estimate separate rate functions for the sub-stages (instars) where differences are observed, as done by Logan et al. [24]. In this case *A* = 0 at the start of each instar, and *A* = 1 (Equation (2)) signals the easily discernible transition between instars (or the transition to the pupal stage). Age dependence *within* the sub-stages (instars) was not investigated. In the case of gypsy moth egg phenology, the transitions between sub-stages (prediapause, diapause, and postdiapause phases) could not be observed. Hence, phase durations could not be measured, and a solution analogous to that for the larval stage (i.e., separate rate functions for the phases) was not available for modeling the age dependence observed by Sanderson and Peairs [23] in gypsy moth egg phenology. Nonetheless, Sawyer et al. [25] modelled the post-diapause induction portion of gypsy moth egg development (diapause and postdiapause, jointly) as an age-dependent process. The Salis et al. [10] data very clearly illustrate an age-dependent developmental response in the egg stage of the winter moth.

### 1.4. Age-Dependent Developmental Response to Temperature within a Life Phase (Sub-Stage)

**Figure 1:**
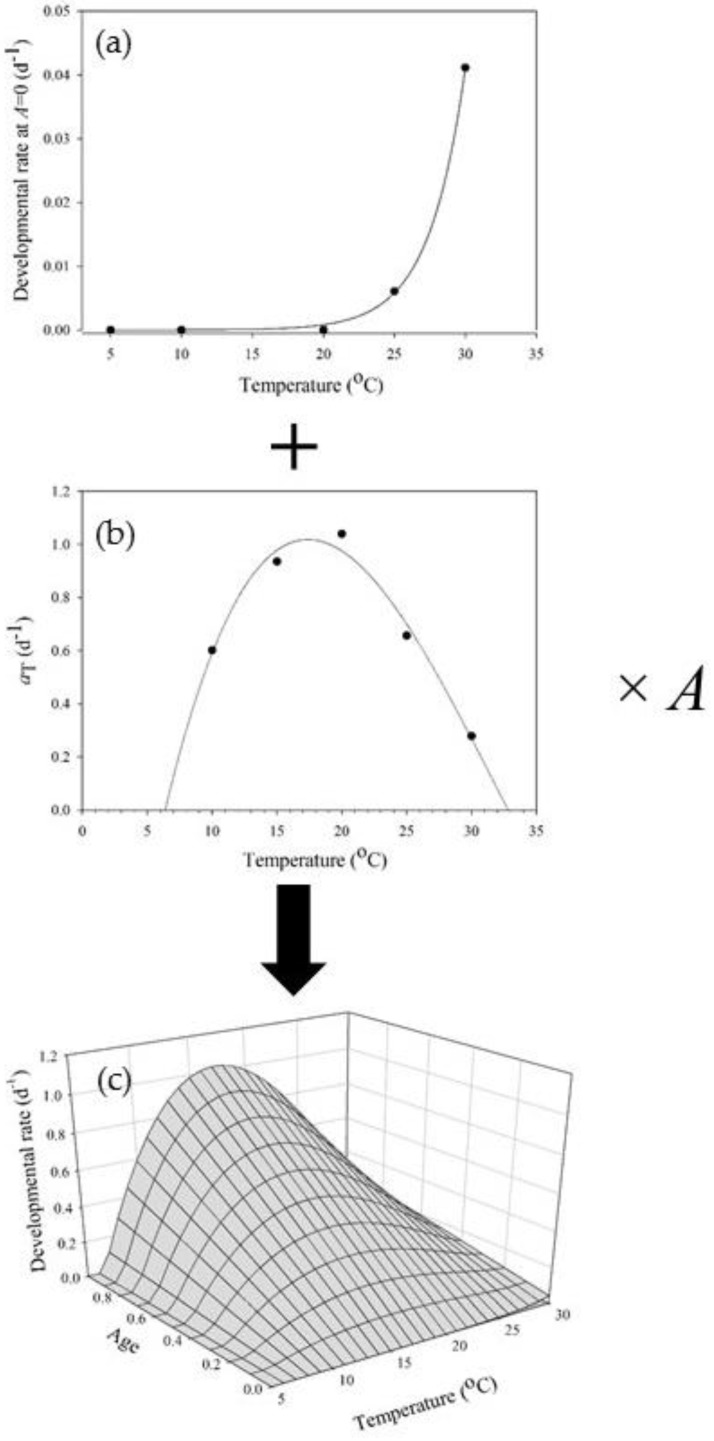
Postdiapause developmental rate in gypsy moth eggs: $r\left(T,A\right)=\tau \times \exp\left(\delta \times T\right)+\left(\omega +\kappa \times T+\psi \times T^{2}+\vartheta \times T^{3}\right)\times A$. Temperature-dependent developmental rates at onset of postdiapause (**a**) increase by a temperature- and age-dependent function (**b**). Postdiapause development is temperature- and age-dependent (**c**). See Gray [28] for derivation of the function and for parameter values.

Gray et al. [26] devised a method of observing the transitions between the developmental phases of the gypsy moth egg stage (prediapause, diapause, and postdiapause). In the gypsy moth egg phenology model of Gray [5], there is a separate rate function for each egg phase (prediapause, diapause, and postdiapause); *A* = 0 at the start of each phase, and *A* = 1 (Equation (2)) signals the transition between phases (or hatch in the case of *A_postdiapause_* = 1). Age dependence in developmental rate was subsequently observed *within* the postdiapause phase [27]. The temperature- and age-dependent postdiapause developmental rate (reformatted by Gray [28]) is estimated by an initial (at *A* = 0) temperature-dependent rate and a temperature-dependent rate change parameter $a_{T}$ (Figure 1):

(7) $$r\left(T,A\right)=r\left(T,A=0\right)+a_{T}\times A.$$

Gray et al. [29] found that the developmental rate function (Equation (3)) also varied over time within the diapause phase of gypsy moth egg development.

An examination of age-dependent developmental rate in winter moth eggs is similar to an examination of the phenomenon in gypsy moth diapause or postdiapause eggs because the smallest discernible developmental unit is examined in each case; age must be dealt with as a continuous variable, not an ordinal (discrete) variable as in the case of instars. The experimental design used by Salis et al. [10] is strikingly similar—albeit with only two experimental temperatures—to the experimental design used by Gray et al. [27,29]. When applied to the Salis et al. [10] data, the analytical method described by Gray et al. [27,28,29] reveals significant disagreements between their model developmental rates and the rates calculated from their experimental observations.

## 2. Materials and Methods

### An Examination of the Winter Moth Egg Phenology Model: Discrepancies between Experimentally-Derived Rates and the Model Predictions

**Figure 2:**
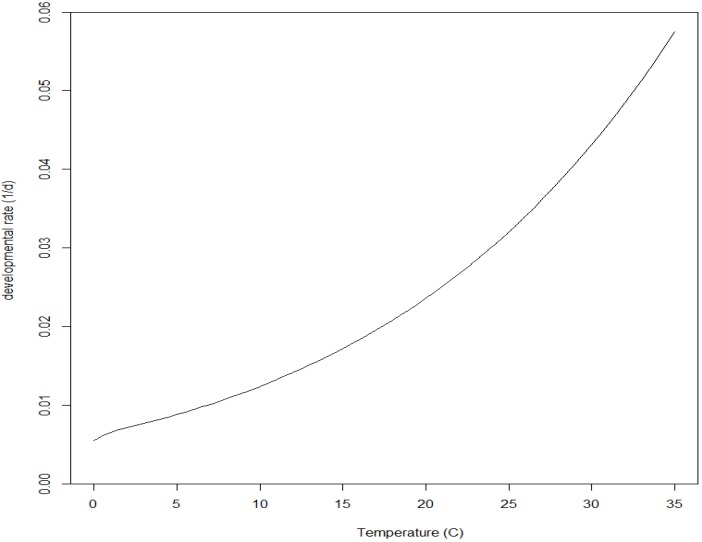
Developmental rate (*r*) vs. temperature (*T*) in the “reduced Sharpe-Schoolfield” model (Equation (5); copied from Salis et al. [10]). $r\left(T\right)=\frac{\frac{\rho T}{T_{ref}}\times \exp\left[\frac{H_{A}}{R}\left(T_{ref}^{-1}-T^{-1}\right)\right]}{1+\exp\left[\frac{H_{L}}{R}\left(T_{L}^{-1}-T^{-1}\right)\right]}$, where $\rho =0.0159\times d^{-1}$, $T_{ref}=286.95^{\circ }\;K$, $H_{A}=42.1\times 10^{3}{\;J\;\mathrm{mol}}^{-1}$, $H_{L}=1216\times 10^{3}{\;J\;\mathrm{mol}}^{-1}$, $T_{L}=272.15^{\circ }\;K$, and *R* is the universal gas constant (8.314 J °K^−1^ mol^−1^).

**Figure 3:**
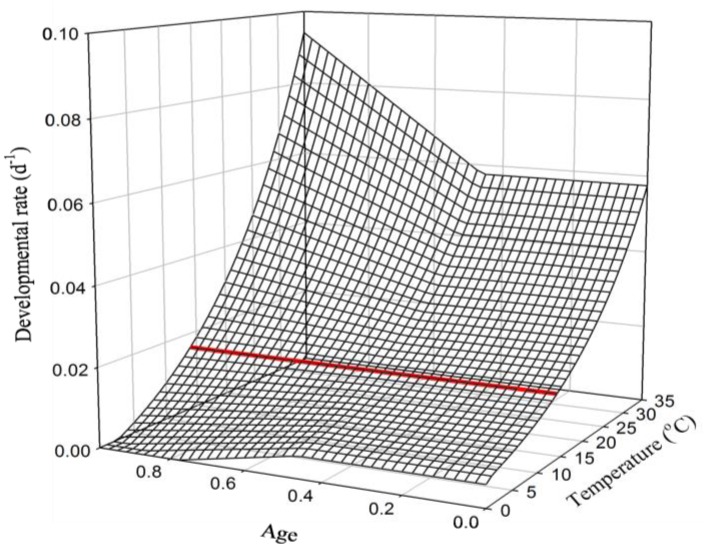
Age-(*A*) and temperature (*T*)-dependent developmental rates from Equation (8) (copied from Salis et al. [10]); the developmental rate at the threshold temperature (13.8 °C, at which the age effect is zero) is displayed in the bold red line. $r\left(T,A\right)=\{\begin{array}{ll} P\left(T\right) & \mathrm{for}\;A<0.46\\ P\left(T\right)+\left(A-0.46\right)\times S\times \left(P\left(T\right)-\rho \right) & \mathrm{for}\;A\geq 0.46 \end{array}$, where Ρ(T) is the rate from Equation (5), *S* is a constant positive scaler (1.63) and *ρ* is from Equation (5) (=developmental rate at *T* = 13.8 °C)

To incorporate age-dependent developmental rates—which Salis et al. [10] detected from their experimental observations of developmental duration—into their phenology model, the authors added a factor to their “reduced Sharpe-Schoolfield” model (Equation (5); Figure 2) whereby developmental rates at ages (Equation (2)) ˃0.46 are reduced at temperatures <13.8 °C and increased at temperatures ˃13.8 °C:

(8) $$r\left(T,A\right)=\{\begin{array}{ll} P(T) & \mathrm{for}\;A<0.46\\ P(T)+\left(A-0.46\right)\times S\times \left(P(T)-\rho \right) & \mathrm{for}\;A\geq 0.46 \end{array}$$

where Ρ(T) is the rate from Equation (5), *S* is a constant positive scaler (1.63) and *ρ* is from Equation (5) (=developmental rate at *T* = 13.8 °C) (Figure 3). Hereafter, I refer to this as the Salis model.

Régnière [30] described the methodology for estimating the developmental rate (*r*) that occurs during a short-term exposure to a given temperature (*T*), as in the Salis et al. [10] experiment. Gray et al. [27,28,29] used the method to describe the changing (i.e., age-dependent) developmental rate response to temperature. If developmental duration is $D_{T_{C}}$ at a constant control temperature $T_{C}$, or the sum of an exposure duration at time *t* to an experimental temperature $T_{E}$ plus the remaining duration at the control temperature (i.e., $d_{t,T_{E}}+d_{T_{C}}$), then the proportion of development that occurs during the exposure to the experimental temperature $T_{E}$ at time *t* is $1-\frac{d_{T_{C}}}{D_{T_{C}}}$, and the developmental rate at the experimental temperature $T_{E}$ during time *t* is

(9) $$r\left(T_{E},t\right)=\frac{1-\frac{d_{T_{C}}}{D_{T_{C}}}}{d_{t,T_{E}}}.$$

Salis et al. [10] have an abundance of field and laboratory data, but only those data summarized in their Figure 1 (available online at Dryad Digital Repository: http://dx.doi.org/10.5061/dryad.8t3v5, “Figure 1. egg-hatching dates experiment 2007”) are from an experiment with the requisite treatments (i.e., known durations at an experimental temperature and at a control temperature) for the estimation of the developmental rate responses to temperature at different times *t*. I used Equation (9) to estimate the developmental rates at the two experimental temperatures $T_{E}=5\;{}^{\circ}C$ and 15 °C during each of the 10 exposure times from the developmental durations of each of the 10–11 families in the Dryad Digital Repository data (“Figure 1. egg-hatching dates experiment 2007”)—hereafter called the observed developmental rates. A mean observed developmental rate was calculated at each combination of experimental temperature and exposure time from the 10 to 11 families. I also calculated the model developmental rates and the accumulated model ages during each exposure to $T_{E}=5\;{}^{\circ}C$ and 15 °C from the Salis model (Equation (8))—hereafter called the model rates and ages, respectively.

## 3. Results

**Figure 4:**
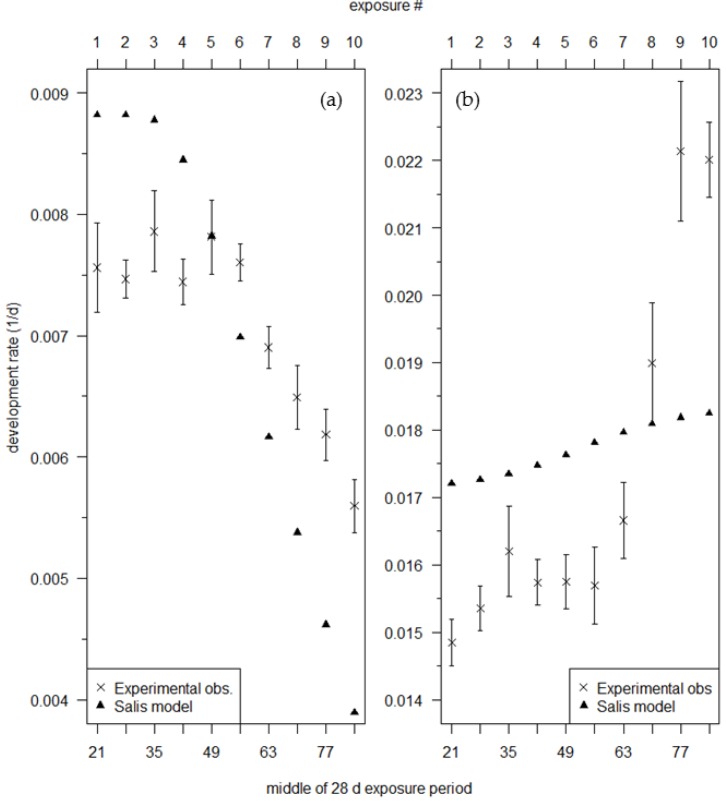
Age-dependent developmental rates during the 28 d exposures to (**a**) 5 °C and (**b**) 15 °C. A model rate (▲) is the mean of the 28 daily rates calculated from the Salis [10] model (Equation (8)). An experimentally observed rate (mean (×) and standard error) is estimated (Equation (9)) from the observed developmental durations of the 10 or 11 families per exposure period. #—number.

At 5 °C, the Salis model (Equation (8)) overestimates developmental rate during the first exposure period by 17% and underestimates developmental rate during the tenth exposure period by 31%. In general, the Salis model overestimates the experimentally observed effect of age on developmental rate at 5 °C by approx. 2.0× (Figure 4a). Observed mean developmental rate at *T* = 5 °C decreased by 26% of its initial rate, from a mean of 0.0076 d^−1^ during the first six exposure periods (where no trend was noticeable) to 0.0056 d^−1^ during the last exposure period. Rates calculated by the Salis model for *T* = 5 °C decrease by 56% of their starting value, from 0.0089 d^−1^ to 0.0039 d^−1^. On the other hand, at 15 °C the model captures only ⅛ of the experimentally observed effect of age on developmental rate (Figure 4b). Observed rates increased by 48% between the first and tenth transfer periods, but modeled rates increase by only 6%. The Salis model overestimates developmental rate at 15 °C during the first seven exposure periods and underestimates developmental rate during the last three exposure periods.

**Figure 5:**
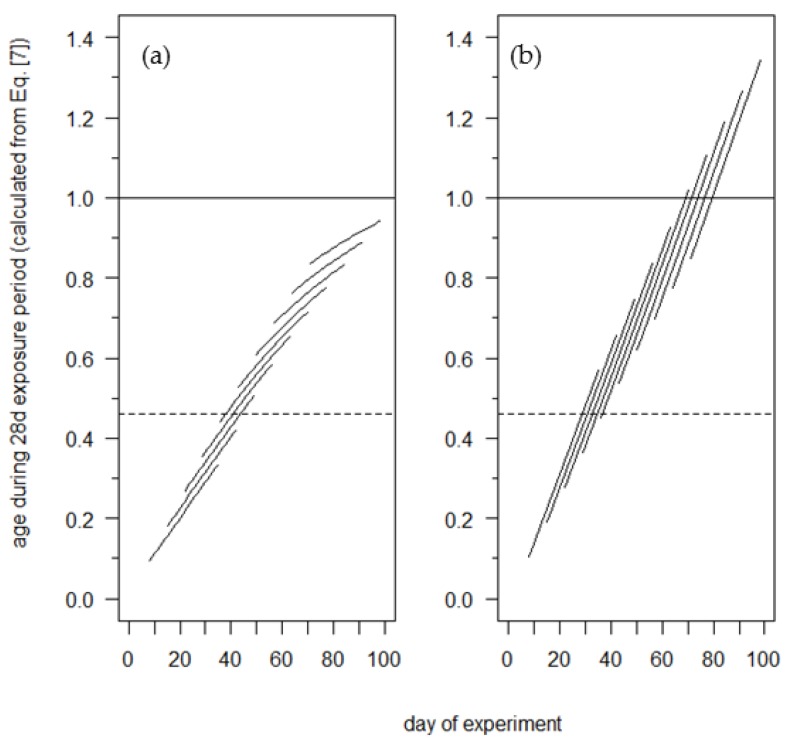
Each solid line illustrates the daily progression of developmental age during a 28 d exposure to (**a**) 5 °C and (**b**) 15 °C calculated with the Salis model (Equation (8)). The dotted line designates the age threshold (*A* = 0.46) above which the Salis model rates decrease (5 °C) or increase (15 °C), and *A* = 1 (hatch).

The age effect of the Salis model exerts its influence on developmental rates earlier than seen in the observed rates at 5 °C. The Salis model rates at 5 °C begin to decline by the fourth exposure period (days 28–55) (Figure 4a) because age passes the *A* = 0.46 threshold of their model during this exposure period (Figure 5a). In contrast, the observed developmental rates do not begin to decline until the seventh exposure period (days 49–76) (Figure 4a). Finally, the Salis model predicts egg hatch (*A =* 1) during the sixth to tenth exposure periods at 15 °C (Figure 5b), but egg hatch was not observed during those exposure periods. The predicted hatch during the eighth to tenth exposure periods are slightly earlier (3–6 d) than observed (Table 1).

## 4. Discussion

The foundation of a phenology model is the rate function, where developmental rate *r* is a function of temperature *T* (Equation (3)) in the classical paradigm, or of *T* and of age *A* (r=f(T,A)). Salis et al. [10] employed an experimental design that very closely resembles the one used by Gray et al. in their investigations of the temperature and age dependence in the developmental rate functions for gypsy moth diapause [29] and postdiapause [27]. However, Salis et al. [10] did not calculate the developmental rates from their developmental duration data that illustrate that the age dependence observed in their data is poorly mimicked by their model (Equation (8)) (Figure 4a,b); their exposure duration was arguably too long for accurate rate estimation in most of the treatments, and they used too few (only two) experimental temperatures.

Salis et al. [10] used a 28-d exposure period to test for an effect of age on the developmental duration of winter moth. This duration of exposure to an experimental temperature ($d_{t,T_{E}}$) is arguably too long for a reasonable estimation of the developmental rate that occurs during an exposure to $T_{E}$ in an age-dependent developmental rate process: as $d_{t,T_{E}}$ becomes shorter, the amount of time at the control temperature ($d_{T_{C}}$) becomes longer, and $1-\frac{d_{T_{C}}}{D_{T_{C}}}$ becomes a more accurate estimate of the amount of development that occurred during the exposure to $T_{E}$ at time *t* (Equation (9)), regardless of the relationship between developmental rate and age [27]. Approx. 20% and 40% of the egg development (arguably too much) occurred during the 28-d exposures to the experimental temperature 5 and 15 °C, respectively (Table 2).

A major benefit of a non-linear temperature-dependent rate function is its ability to capture the increasing and decreasing developmental rates that occur below and above the optimum temperature, respectively. Salis et al. [10] did not use experimental temperatures above the likely optimum at which a declining developmental rate occurs, and so removed two parameters for their “reduced Sharpe-Schoolfield” model (Equation (5); Figure 2). However, with only two experimental temperatures it is impossible to assume any particular non-linear functional form and these data provide no justification for selecting such a parameter-heavy (five parameters) nonlinear model over a more parsimonious formulation to which the same age-dependent factor could be added.

Salis et al. [10] noted that their model predictions of winter moth egg hatch dates are “generally later than the observed [dates from field data]”. The authors suggest several possible reasons for the discrepancy, but more or less discount each. The developmental rates I have calculated from the Salis et al. [10] model (Equation (8)) do not adequately mimic their data: their model rates deviate substantially from the age-dependent developmental responses calculated from their data (Figure 4a,b). In particular, the model underestimates the observed rates at 15 °C during the later exposure periods which are close to the time of hatch when temperature effects are the strongest [10]. Presumably, actual rates at temperatures further above the authors’ model threshold (13.8 °C) are similarly—or more severely—underestimated. This underestimation of developmental rates in the late portion of egg development is a likely cause of the discrepancy noted by the authors.

The authors also question whether the 24-h time step of their model—using daily mean temperatures as input—is too long to capture the effect of diurnal temperature variation on developmental progress. They justify their choice of time step by citing Wagner et al. [31], but Wagner et al. [31] (p. 208) say only that “mean daily rates *can* be accumulated under fluctuating temperature environments” (emphasis mine); nowhere do they discuss the implications of using mean daily temperatures as inputs to a nonlinear rate function. In fact, this issue should be of considerable interest to biologists using a phenology model to predict the time of an event. Jensen’s inequality states that f(E[x])≤E[f(x)] if f is a convex function. Restated in terms relevant to phenology modeling, it tells us that

(10) $$r\left(\bar{T}\right)\leq \sum_{i=1}^{2}\frac{r\left(T_{i}\right)}{2}\;\mathrm{when}\;\frac{d^{2}r}{dT^{2}}>0\;\mathrm{for}\;i=\left\{T_{\min},T_{\max}\right\}.$$

**Figure 6:**
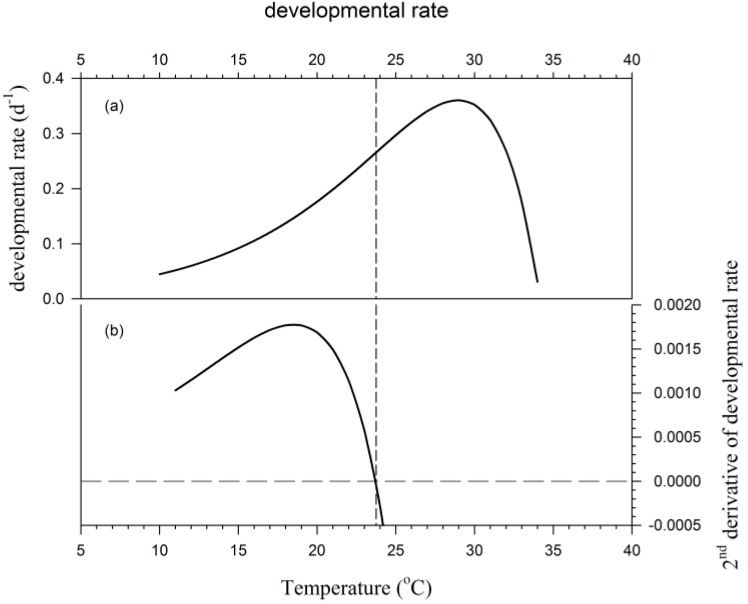
The vertical line indicates the temperature at which the second derivative (**b**) of a rate function (**a**) equals zero. Below this temperature, modeled daily development is less when using mean daily temperatures than when using daily minimum and maximum, or hourly temperatures. The reverse is true above this temperature.

That is, modeled phenological development for a day (24 h) is less if the mean daily temperature is used in a 24-h time step than it is if the daily minimum and maximum temperatures are used in a 12-h time step when the temperatures are in the portion of the rate curve where the second derivative is positive. The reverse is true when $\frac{d^{2}r}{dT^{2}}<0\;\mathrm{for}\;i=\left\{T_{\min},T_{\max}\right\}$ (Figure 6). The same rules apply if comparing the use of mean daily to hourly temperatures, etc. The computing speed of today’s desktop computers makes it unnecessary to accept the less-accurate simulations generated with mean daily temperatures.

## 5. Conclusions

The factors that determine a species’ global distribution are numerous, multi-trophic, and not completely understood, but climate has long been recognized as a dominant factor [32]. Range expansions/contractions of several insect groups have been associated with recent climate trends [33,34,35,36,37,38,39]. Most predictions of future distributions—under projected future climates—rely on a correlative technique in a niche model wherein the observed presence of a species is associated with some number of climate variables, such as minimum winter temperature, maximum summer temperature, summer degree-days, etc., and the spatial occurrence of those climate variables is estimated in the future climate scenario. Limitations to such a technique have been described by several authors [40,41,42,43,44,45]. A different niche model technique [46] relies on a phenology model to test the likelihood that the climate does now—or will in a future climate scenario—produce an “appropriate seasonality” [15] for the species. For temperate insects, an appropriate seasonality is one in which larvae (the feeding stage) emerge when a suitable food source is available, they are in a cold-hardy stage when winter occurs, and seasonal temperature variation satisfies their developmental requirements each year. Gray [5] demonstrated the technique with the gypsy moth in North America. The irrefutable non-linearity of the developmental rate curve means that, at some point, further increases in temperature will have a negative impact on distribution. The analysis by Tobin et al. [47] demonstrated a strong connection between frequent supraoptimal temperatures and range retraction in the gypsy moth.

In general, reliable estimates of a potential range rely on two conditions: (1) a geographically robust [48] phenology model must exist; and (2) bias from simulation procedures must be avoided [49]. Gray [5] defined the “geographic robustness” of a phenology model as “the ability to perform satisfactorily over a broad geographic range”. The timing of egg hatch is arguably the most critical phenological event in the life-cycle of temperate insects. The history of egg hatch phenology models in the gypsy moth [25,50,51] and the winter moth [11,12,13,14] illustrate the difficulty of achieving geographic robustness. Geographic robustness was achieved for gypsy moth egg hatch when development in diapause and postdiapause were modelled as temperature- and age-dependent [27,28,29] processes. The work of Salis et al. [10] is important, not as “novel experimental findings” [10] (p. 1777), but because it clearly demonstrates an age-dependent component to the developmental rate process in the egg stage of another insect family. The near-universal assumption of age-independence in phenology models is rarely tested [24]. The earlier work of Gray et al. [27,29], and now that of Salis et al. [10], should provide a strong justification for further testing of this basic assumption. Incorporating age-dependence into a phenology model where it can be experimentally demonstrated will improve the geographic robustness of phenological and range predictions.

## Figures and Tables

**Table 1:** Comparison of the hatch day predicted by the Salis model and experimentally observed in the 15 °C treatments.

Exposure #	Exposure Period (d) ^1^	Predicted Hatch Day ^2^	Observed Hatch Day ^3^
6	42–70	69	na
7	49–77	71	na
8	56–84	74	80
9	63–91	77	80
10	70–98	80	85

#—number. **^1^** Hatch occurred in some families before the end of the prescribed exposure period. **^2^** Predicted from Equation (8). **^3^** Median (50% cumulative) hatch day derived from the recorded hatch day for each of the 10 or 11 families in each treatment.

**Table 2:** Estimated proportion of total development that occurred during the exposures to experimental temperatures (=1−dTCDTC; as described for Equation (9)).

Exposure #	Exposure Period (d)	Development at 5 °C	Development at 15 °C
1	7–35	0.21	0.42
2	14–42	0.21	0.43
3	21–49	0.22	0.45
4	28–56	0.21	0.44
5	35–63	0.22	0.44
6	42–70	0.21	0.44
7	49–77	0.19	0.47
8	56–84 ^1^	0.18	0.46
9	63–91 ^1^	0.17	0.40
10	70–98 ^1^	0.16	0.33

^1^ Egg hatch occurred in some families before the scheduled 28-d exposure period was completed. The proportions are calculated for the actual exposure durations.

## References

[1] Chen X., Schwartz M.D. (2003). Phenological Data, Networks, and Research: East Asia. Phenology: An Integrative Environmental Science.

[2] Réaumur R.A.F. (1735). Obserations du thermomètre, Faites à Paris Pendant L’année 1735, Comparées Avec Celles Qui ont été Faites Sous la Ligne, à l’isle de France, à Alger et Quelques Unes de nos Isles de l’Amérique.

[3] Damos P.T., Savopoulou-Soultani M. (2010). Development and statistical evaluation of models in forecasting moth phenology of major lepidopterous peach pest complex for Integrated Pest Management programs. Crop Prot..

[4] Moerkens R., Gobin B., Peusen G., Helsen H., Hilton R., Dib H., Suckling D.M., Leirs H. (2011). Optimizing biocontrol using phenological day degree models: The European earwig in pipfruit orchards. Agric. For. Entomol..

[5] Gray D.R. (2004). The gypsy moth life stage model: Landscape-wide estimates of gypsy moth establishment using a multi-generational phenology model. Ecol. Model..

[6] Logan J.A., Réqnière J., Gray D.R., Munson A.S. (2007). Risk assessment in face of a changing environment: Gypsy moth and climate change in Utah. Ecol. Appl..

[7] Gray D.R. (2015). Risk reduction of an invasive insect by targeting surveillance efforts with the assistance of a phenology model and international maritime shipping routes and schedules. Risk Anal..

[8] Gray D.R. (2016). Climate change can reduce the risk of biological invasion by reducing propagule pressure. Biol. Invasions.

[9] Van Asch M., Visser M.E. (2007). Phenology of forest caterpillars and their host trees: The importance of synchrony. Ann. Rev. Entomol..

[10] Salis L., Lof M., van Asch M., Visser M.E. (2016). Modeling winter moth *Operophtera brumata* egg phenology: Nonlinear effects of temperature and developmental stage on developmental rate. Oikos.

[11] Embree D.G. (1970). The diurnal and seasonal pattern of hatching of winter moth eggs, *Operophtera brumata* (Geometridae: Lepidoptera). Can. Entomol..

[12] Kimberling D.N., Miller J.C. (1988). Effects of temperature on larval eclosion of the winter moth, *Operophtera brumata*. Entomol. Exp. Appl..

[13] Visser M.E., Holleman L.J.M. (2001). Warmer springs disrupt the synchrony of oak and winter moth phenology. Proc. R. Soc. B Biol. Sci..

[14] Hibbard E.L., Elkinton J.S. (2015). Effect of spring and winter temperatures on winter moth (Geometridae: Lepidoptera) larval eclosion in the Northeastern United States. Environ. Entomol..

[15] Régnière J., Logan J.A., Schwartz M.D. (2003). Animal life cycle models. Phenology: An Integrative Environmental Science.

[16] Schwartz M.D., Schwartz M.D. (2003). Introduction. Phenology: An Integrative Environmental Science.

[17] Chuine I., Régnière J. (2017). Process-Based Models of Phenology for Plants and Animals. Ann. Rev. Ecol. Evol. Syst..

[18] Egerton F.N. (2006). A history of the ecological sciences, part 21: Réaumur and his history of insects. Bull. Ecol. Soc. Am..

[19] Janisch E. (1932). The influence of temperature on the life-history of insects. Ecol. Entomol..

[20] Sharpe P.J.H., Demichele D.W. (1977). Reaction kinetics of poililotherm development. J. Theor. Biol..

[21] Schoolfield R.M., Sharpe P.J.H., Magnuson C.E. (1981). Non-linear regression of biological temperature-dependent rate models based on absolute reaction-rate theory. J. Theor. Biol..

[22] Sanderson E.D. (1910). The relation of temperature to the growth of insects. J. Econ. Entomol..

[23] Sanderson E.D., Peairs L.M. (1913). The relation of temperature to insect life. Technical Bulletin.

[24] Logan J.A., Casagrande P.A., Liebhold A.M. (1991). Modeling environment for simulation of gypsy moth (Lepidoptera: Lymantriidae) larval phenology. Environ. Entomol..

[25] Sawyer A.J., Tauber M.J., Tauber C.A., Ruberson J.R. (1993). Gypsy moth (Lepidoptera: Lymantriidae) egg development: A simulation analysis of laboratory and field data. Ecol. Model..

[26] Gray D.R., Logan J.A., Ravlin F.W., Carlson J.A. (1991). Toward a model of gypsy moth egg phenology: Using respiration rates of individual eggs to determine temperature-time requirements of prediapause development. Environ. Entomol..

[27] Gray D.R., Ravlin F.M., Régnière J., Logan J.A. (1995). Further advances toward a model of gypsy moth (*Lymantria dispar* (L.)) egg phenology: Respiration rates and thermal responsiveness during diapause, and age-dependent developmental rates in postdiapause. J. Insect Physiol..

[28] Gray D.R. (2009). Age-dependent postdiapause development in the Gypsy Moth (Lepidoptera: Lymantriidae) Life Stage Model. Environ. Entomol..

[29] Gray D.R., Ravlin F.W., Braine J.A. (2001). Diapause in the gypsy moth: A model of inhibition and development. J. Insect Physiol..

[30] Régnière J. (1987). Temperature-dependent development of eggs and larvae of *Choristoneura fumiferana* (Clem.) (Lepidoptera: Tortricidae) and simulation of its seasonal history. Can. Entomol..

[31] Wagner T.L., Wu H.-I., Sharpe P.J.H., Schoolfield R.M., Coulson R.N. (1984). Modeling insect development rates: A literature review and application of a biophysical model. Ann. Entomol. Soc. Am..

[32] Andrewartha H.G., Birch L.C. (1954). The Distribution and Abundance of Animals.

[33] Bale J.S., Masters G.J., Hodkinson I.D., Awmack C., Bezemer T.M., Brown V.K., Butterfield J., Buse A., Coulson J.C., Farrar J. (2002). Herbivory in global climate change research: Direct effects of rising temperature on insect herbivores. Glob. Chang. Biol..

[34] Parmesan C., Ryrholm N., Stefanescu C., Hill J.K., Thomas C.D., Descimon H., Huntley B., Kaila L., Kullberg J., Tammaru T. (1999). Poleward shifts in geographical ranges of butterfly species associated with regional warming. Nature.

[35] Battisti A., Stastny M., Buffo E., Larsson S. (2006). A rapid altitudinal range expansion in the pine processionary moth produced by the 2003 climatic anomaly. Glob. Chang. Biol..

[36] Crozier L. (2004). Warmer winters drive butterfly range expansion by increasing survivorship. Ecology.

[37] Jepsen J.U., Hagen S.B., Yoccoz N.G. (2008). Climate change and outbreaks of the geometrids *Operophtera brumata* and *Epirrita autumnata* in subarctic birch forest: Evidence of a recent outbreak range expansion [electronic resource]. J. Anim. Ecol..

[38] Wilson R.J., Hagen S.B., Ims R.A., Yoccoz N.G. (2005). Changes to the elevational limits and extent of species ranges associated with climate change. Ecol. Lett..

[39] Parmesan C. (2006). Ecological and evolutionary responses to recent climate change. Ann. Rev. Ecol. Evol. Syst..

[40] Araújo M.B., Guisan A. (2006). Five (or so) challenges for species distribution modelling. J. Biogeogr..

[41] Davis A.J., Jenkinson L.S., Lawton J.H., Shorrocks B., Wood S. (1998). Making mistakes when predicting shifts in species range in response to global warming. Nature.

[42] Barry S., Elith J. (2006). Error and uncertainty in habitat models. J. Appl. Ecol..

[43] Buisson L., Thuiller W., Casajus N., Lek S., Grenouillet G. (2010). Uncertainty in ensemble forecasting of species distribution. Glob. Chang. Biol..

[44] Heikkinen R.K., Louto M., Araújo M.B., Virkkala R., Thuiller W., Sykes T. (2006). Methods and uncertainties in bioclimatic envelope modelling under climate change. Prog. Phys. Geogr..

[45] Thuiller W., Brotons L., Araújo M.B., Lavorel S. (2004). Effects of restricting environmental range of data to project current and future species distributions. Ecography.

[46] Chuine I. (2010). Why does phenology drive species distribution?. Philos. Trans. R. Soc. B Biol. Sci..

[47] Tobin P.C., Gray D.R., Liebhold A.M. (2014). Supraoptimal temperatures influence the range dynamics of a non-native insect. Divers. Distrib..

[48] Gray D.R., Zhang X. (2012). Using geographically robust models of insect phenology in forestry. Phenology and Climate Change.

[49] Gray D.R. (2014). Unwanted spatial bias in predicting establishment of an invasive insect based on simulated demographics. Int. J. Biometeorol..

[50] Johnson P.C., Mason D.P., Radke S.L., Tracewski K.T. (1983). Gypsy moth, *Lymantria dispar* (L.) (Lepidoptera: Lymantriidae), egg eclosion: Degree-day accumulation. Environ. Entomol..

[51] Lyons D.B., Lysyk T.J., Wallner W.E., McManus K.A. (1989). Development and phenology of eggs of gypsy moth *Lymantria dispar* (Lepidoptera: Lymantriidae) in Ontario. Proceedings, Lymantriidae: A Comparison of Features of New and Old World Tussock Moths.

